# Within-task variability on standardized language tests predicts autism spectrum disorder: a pilot study of the Response Dispersion Index

**DOI:** 10.1186/s11689-019-9283-z

**Published:** 2019-09-13

**Authors:** Abby E. Hare-Harris, Marissa W. Mitchel, Scott M. Myers, Aaron D. Mitchel, Brian R. King, Brittany G. Ruocco, Christa Lese Martin, Judy F. Flax, Linda M. Brzustowicz

**Affiliations:** 1 Department of Biological and Allied Health Sciences, Hartline Science Center, Bloomsburg University, 400 East Second St, Bloomsburg, PA 17815 USA; 2 Autism & Developmental Medicine Institute, Geisinger Health System, 120 Hamm Drive, Suite 2A, Lewisburg, PA 17837 USA; 3 Psychology Department, O’Leary Center, Bucknell University, Lewisburg, PA 17837 USA; 4 Computer Science Department, Breakiron Building, Bucknell University, Lewisburg, PA 17837 USA; 5 Genetics Department, Life Sciences Building, Rutgers University, 145 Bevier Road, Piscataway, NJ 08854 USA

**Keywords:** Developmental difference, Autism spectrum disorder, Language impairment, Intra-subtest scatter

## Abstract

**Background:**

Qualitatively atypical language development characterized by non-sequential skill acquisition within a developmental domain, which has been called developmental *deviance* or *difference*, is a common characteristic of autism spectrum disorder (ASD). We developed the Response Dispersion Index (RDI), a measure of this phenomenon based on intra-subtest scatter of item responses on standardized psychometric assessments, to assess the within-task variability among individuals with language impairment (LI) and/or ASD.

**Methods:**

Standard clinical assessments of language were administered to 502 individuals from the New Jersey Language and Autism Genetics Study (NJLAGS) cohort. Participants were divided into four diagnostic groups: unaffected, ASD-only, LI-only, and ASD + LI. For each language measure, RDI was defined as the product of the total number of test items and the sum of the weight (based on item difficulty) of test items missed. Group differences in RDI were assessed, and the relationship between RDI and ASD diagnosis among individuals with LI was investigated for each language assessment.

**Results:**

Although standard scores were unable to distinguish the LI-only and ASD/ASD + LI groups, the ASD/ASD + LI groups had higher RDI scores compared to LI-only group across all measures of expressive, pragmatic, and metalinguistic language. RDI was positively correlated with quantitative ASD traits across all subgroups and was an effective predictor of ASD diagnosis among individuals with LI.

**Conclusions:**

The RDI is an effective quantitative metric of developmental deviance/difference that correlates with ASD traits, supporting previous associations between ASD and non-sequential skill acquisition. The RDI can be adapted to other clinical measures to investigate the degree of difference that is not captured by standard performance summary scores.

**Electronic supplementary material:**

The online version of this article (10.1186/s11689-019-9283-z) contains supplementary material, which is available to authorized users.

## Background

In the early twentieth century, Gesell et al. observed that typical development is methodical, sequential, timed, and therefore largely predictable [1, 2]. This principle is the basis for using developmental milestones and tests as markers of neuromaturation. Gesell’s emphasis on patterns and sequences of development set the stage for Piaget, who also emphasized the importance of the *sequence* of staged maturation, while recognizing the role of active experience and social interaction in development [3]. Developmental delay is defined by acquisition of skills in the typical sequence but at a slower rate and, in many cases, with a lower overall developmental ceiling. Delay within a developmental domain results in a measurable performance deficit relative to age norms, and early childhood deficits may be temporary or they may persist. Because the sequence of skill attainment is preserved, developmental delay results in a profile of abilities within one or more developmental domains that is similar to the performance of a younger typically developing child. Conversely, the concept of qualitatively atypical development characterized by non-sequential skill acquisition within a developmental domain (with or without developmental delay), resulting in a more widely scattered profile of ability, has been described as *developmental deviance* [4–6] or *developmental difference* [7]. In this case, a child attains more difficult skills within a developmental sequence without having accomplished easier tasks in the sequence. In contrast to developmental delay, this pattern of development results in a profile of abilities that is not commonly found in typically developing children of any age. Although developmental delay is routinely quantified and represented by a rate, quotient, or standard score, atypicality due to non-sequential development is typically described qualitatively [5, 6]. Despite the emphasis on the importance of the sequence of developmental progression by Gesell and Piaget, among others, it remains largely unmeasured in clinical practice. The phenomenon of non-sequential, qualitatively atypical development is manifested on psychometric tests as unusual or inconsistent response patterns to items within subtest scales, resulting in increased intra-subtest scatter or within-task variability [8–10]. It would be useful to quantify this phenomenon to allow investigation of its utility for making clinical diagnoses, prognosticating, and advancing understanding of typical and disordered development.

Qualitative atypicality of language development is a prominent characteristic of autism spectrum disorder (ASD). For example, atypical sequences of skill attainment in the areas of communication and socialization have been reported in individuals with ASD [11]. Echolalia, jargon, and other unusual, non-developmental semantic and syntactic error patterns are also more frequent among children with ASD than typically developing children or children with intellectual disability [12, 13]. Even among individuals with ASD who have higher verbal skills, the language profile that emerges in childhood and persists into adulthood is characterized by unevenness, including poor comprehension relative to apparent expressive language abilities, semantic processing anomalies despite normal performance on vocabulary tests, and idiosyncratic word usage despite relatively intact articulation and syntax [14]. This pattern of language development that is often observed in individuals with ASD is generally thought to be distinct from that found in developmental language disorder [15, 16]. Although “developmental language disorder” (DLD) is currently the preferred term for unexplained language problems in children, much of the pertinent research has focused on “specific language impairment” (SLI) [17]. Unlike DLD, the diagnosis of SLI traditionally required a normal non-verbal IQ and, in some cases, a large discrepancy between verbal and non-verbal ability (a criterion lacking in validity) [17]. Because much of the past research used the SLI definition, we have maintained the use of the term “SLI” when referring to those studies, although the conclusions likely apply to the current “DLD” terminology. SLI, defined as persistent deficits in language skills in the absence of broader cognitive impairment or hearing loss, affects up to 7% of young children [18]. In contrast to ASD, SLI is not clearly associated with prominent non-sequential milestone attainment, and children with SLI can be viewed as exhibiting more of a delayed pattern of development [19, 20]. Despite the distinctions between ASD and SLI, there is also a notable overlap in the language profiles of some individuals with SLI and ASD, particularly in pragmatic language difficulties [21, 22] and structural language impairments [23–26]. Furthermore, language development in preschool children with ASD is frequently observed to be qualitatively atypical as well as delayed [27]. These observations highlight the heterogeneity of both disorders and the potential value of a quantitative metric for assessment of non-sequential skill acquisition for understanding language development and distinguishing among clinical disorders.

**Fig. 1:**
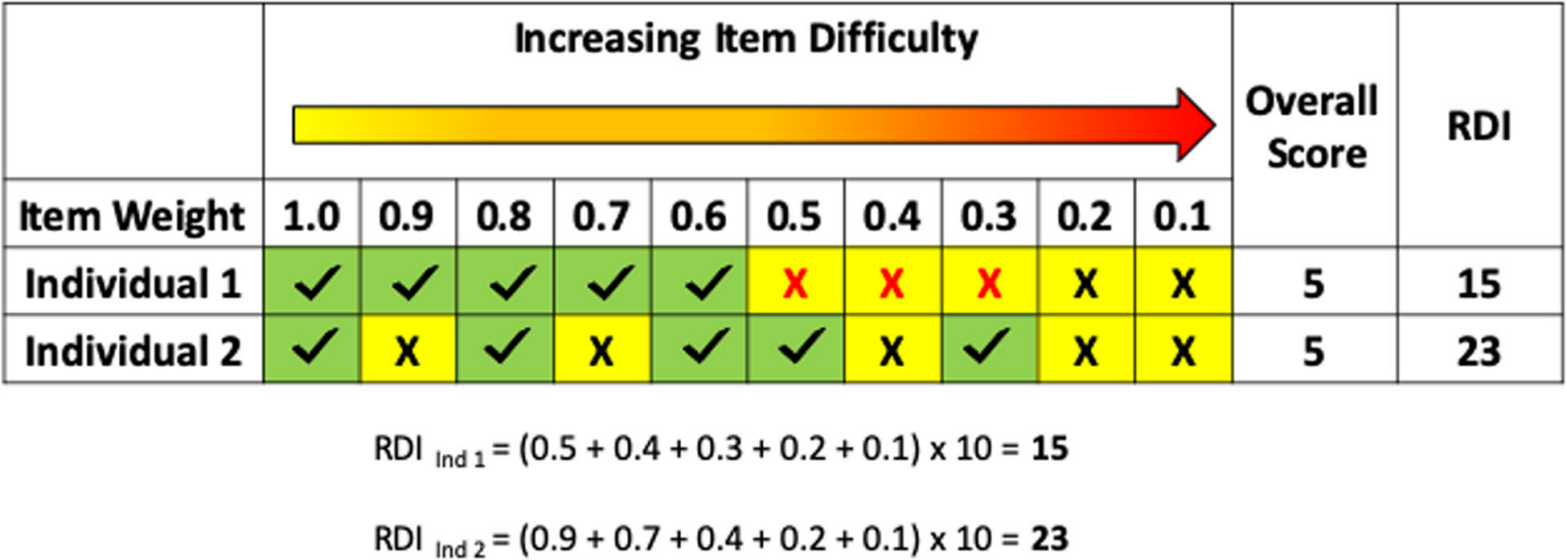
Distribution of incorrect responses to a clinical measure that is arranged hierarchically and demonstration of RDI calculation. Individual 1 represents a participant who displays developmental delay and reaches a testing ceiling (depicted with red X’s). Individual 2 represents a participant who displays developmental difference and does not reach a testing ceiling. The overall score is the total number of correct responses (check marks). Weights for each test item are defined as the percentage of typically developing individuals who responded correctly. The Response Dispersion Index (RDI) calculation is shown for individuals 1 and 2

Standardized psychometric tests are routinely used in clinical practice to evaluate cognition, language, and other aspects of development. Although these metrics are effective for identifying specific developmental delays and persistent deficits, performance summary scores do not capture atypical response patterns indicative of non-sequential development [8]. Many standardized assessments are arranged hierarchically, using a series of items of graded difficulty. An individual’s raw score is determined by establishing a basal, or initial sequence of a certain number of correct answers, and a ceiling, based on a stoppage rule (e.g., a certain number of consecutive items answered incorrectly). Typically developing individuals and those with developmental delay are expected to pass all items to a level of maximum capacity and then fail all items beyond that point (ceiling), with some normal variability occurring around the items at the approximate ability level of the individual being tested [8]. However, individuals with non-sequential development exhibit a more scattered pattern of incorrect answers that is not captured in the standard score. As illustrated in Fig. 1, two individuals with the same overall score (number of correct responses) on a particular subtest may appear to be impaired to a similar extent in that domain; however, at the item response level, the distribution of incorrect answers within a subtest might tell a different story. Increased within-task variability, or intra-subtest scatter, could result from answering some of the easier items incorrectly while answering harder items correctly (as in Fig. 1) or a tendency toward failure on individual items after long runs of correct responses. The degree of dispersion of correct responses, or within-task variability, warrants investigation as a means of measuring aspects of performance that cannot be captured by currently available standardized subtest performance scores.

Several measures of within-task variability have been applied to clinical intelligence metrics as indicators of cognitive dysfunction, with mixed results in distinguishing individuals with various brain insults or psychiatric disorders from controls [8, 28]. Although few studies have assessed within-task variability on direct measures of language ability quantitatively, one study found that response patterns for children with ASD differed significantly from those of typically developing children and children with non-specific developmental delays on several measures of syntactic development [13]. In this pilot study, we quantified atypical language development resulting from non-sequential skill acquisition by measuring dispersion of item-level responses (within-task variability) on standardized language measures and examined this metric in groups of individuals with ASD with and without language impairment (LI), individuals with LI alone, and controls without ASD or LI. We further assessed the association between within-task variability and the degree of quantitative ASD traits as measured by the Social Responsiveness Scale (SRS) [29].

## Methods

### Participants

This study is a retrospective study of 502 individuals (154 families) from the New Jersey Language and Autism Genetics Study (NJLAGS) cohort [30–32]. All NJLAGS families were initially ascertained for at least one individual with ASD (with no known genetic cause), at least one other family member with a language impairment, and additional unaffected family members willing to participate. All participants (mean age = 52 years old; 53% male), regardless of family membership, were included in this study as individual subjects. The average age of participants with ASD was 13 years old, and the average age of those with language impairment was 24 years old. Participants were primarily recruited from the New Jersey area with the following ethnicity breakdown: 75% White/Caucasian, 5% African American, 1% Asian, < 1% Pacific Islander, 6% more than one race, and 12% unknown/unspecified. Since all language measures are standardized for the English language, all participants were required to have English as their primary language. The study was approved by the Rutgers University Institutional Review Board and the Institutional Review Boards at Geisinger Health System and Bloomsburg University.

### Assessments

All individuals were evaluated using a comprehensive testing battery that included measures of oral language, autistic traits, and cognitive ability. Six subtests of the Clinical Evaluation of Language Fundamentals, Fourth Edition (CELF-4) [33, 34] were included: Word Structure, Recalling Sentences, Formulating Sentences, Word Classes (Expressive and Receptive), Concepts and Following Directions, and Word Definitions. Five subtests of the Comprehensive Assessment of Spoken Language (CASL) [35] were included in the testing battery: Non-literal Language, Ambiguous Sentences, Meaning from Context, Pragmatic Judgment, and Inference. All assessments were administered using standard basal and ceiling rules. Standard scores for the individuals > 21 years old were determined using the normative values for individuals who are 21 years old. Language impairment (LI) was defined as having a core standard score of ≤ 85 on the CELF-4. Alternatively, an individual without a CELF-4 core standard score qualified as having LI if he/she scored at least 1 SD below the standard mean on 60% of all language measure subtests administered based on age of subject. A total of 19 individuals were removed for having > 10% missing data.

All individuals with ASD met criteria for autism on the following measures: (1) Autism Diagnostic Interview–Revised [36], (2) Autism Diagnostic Observation Schedule [37], and (3) DSM-IV criteria. The autistic traits of all participants, including individuals without ASD, were assessed using the SRS [29]. The SRS is a 65-item questionnaire that assesses the severity of ASD traits using a quantitative scale.

### Diagnostic groups

Since the language tests used in the present study were normed on a pediatric population (up to 21 years old), we focused our phenotypic analyses on individuals ≤ 21 years old (total *n* = 187). These individuals were assigned to four diagnostic groups: (1) individuals with an ASD diagnosis, who did not meet criteria for LI (ASD-only; *n* = 17); (2) individuals who met criteria for LI, but did not have an ASD diagnosis (LI-only; *n* = 28); (3) individuals with an ASD diagnosis who also met criteria for LI (ASD + LI; *n* = 27); and (4) individuals who did not meet criteria for LI or ASD (unaffected; *n* = 115). We also assessed group differences when the cohort was stratified by ASD diagnosis. There were 44 individuals with an ASD diagnosis (ASD+) and 133 without an ASD diagnosis (ASD−). Means and standard deviations for all subtests, IQ, and age for individuals ≤ 21 years old are listed in Table 1.

**Table 1:** Means and standard deviation of standard scores for CELF and CASL subtests

Test		Unaffected		ASD-only		LI-only		ASD + LI	
		*N* < 21	Mean (SD)	*N* < 21	Mean (SD)	*N* < 21	Mean (SD)	*N* < 21	Mean (SD)
		*N* all		*N* all		*N* all		*N* all	
CELF	Concepts and Following Directions	68	10.31 (2.47)	9	10.56 (2.19)	25	4.60 (2.52)	21	3.57 (2.84)
	Formulated Sentences	108	11.33 (2.30)	17	9.71 (2.34)	28	5.43 (2.92)	29	4.07 (3.32)
		332	11.51 (2.17)	18	9.72 (2.27)	46	5.46 (3.01)	32	3.91 (3.25)
	Recalling Sentences	115	10.53 (2.44)	19	8.16 (2.81)	27	5.33 (2.11)	32	3.50 (3.17)
		348	10.04 (2.62)	20	7.85 (3.07)	47	4.77 (2.47)	35	3.46 (3.15)
	Word Structure	34	9.71 (2.34)	6	8.67 (2.50)	16	4.19 (2.56)	11	4.27 (3.32)
	Word Definitions	45	12.62 (2.52)	9	11.56 (1.81)	3	5.67 (0.58)	10	5.20 (4.37)
		278	12.96 (2.31)	9	11.56 (1.81)	23	7.61 (3.65)	13	5.08 (4.25)
	Word Classes-Total	78	11.44 (2.70)	12	9.92 (1.68)	12	6.08 (1.93)	19	5.37 (3.37)
		311	11.67 (2.13)	13	9.31 (2.72)	31	6.26 (2.56)	22	5.00 (3.32)
CASL	Ambiguous Sentences	59	102.20 (15.30)	9	90.22 (19.27)	7	80.14 (6.44)	10	77.50 (12.42)
		286	97.73 (13.68)	10	87.90 (19.60)	25	75.12 (8.82)	13	76.77 (13.92)
	Inference	67	101.37 (11.30)	7	83.29 (24.20)	12	78.50 (16.51)	19	70.58 (20.61)
		70	101.17 (11.75)	7	83.29 (24.20)	12	78.50 (16.51)	19	70.58 (20.61)
	Pragmatic Judgment	115	103.17 (10.88)	21	88.29 (16.70)	27	79.33 (12.96)	28	65.93 (19.75)
		343	98.21 (11.09)	22	86.09 (19.28)	47	79.77 (14.61)	31	64.97 (20.28)
	Non-literal Language	85	106.01 (12.92)	13	96.08 (17.11)	17	85.53 (14.52)	22	70.68 (21.22)
		311	98.98 (13.84)	14	92.07 (22.25)	36	77.56 (17.07)	25	68.80 (21.85)
	Meaning from Context	56	103.46 (18.54)	9	95.44 (15.88)	7	83.00 (7.26)	8	72.00 (17.67)
		283	102.19 (13.17)	10	91.00 (20.53)	25	79.36 (11.90)	11	97.27 (18.47)
Age	Years	115	10.94 (4.55)	17	11.06 (5.89)	28	8.59 (3.59)	27	9.92 (4.00)
		369	29.73 (19.17)	24	30.08 (18.55)	53	27.83 (16.80)	37	28.00 (18.72)
SRS	Total raw score	76	26.36 (24.31)	13	77.85 (31.92)	15	57.93 (38.06)	23	90.13 (34.04)
		279	30.09 (25.45)	20	87.00 (34.69)	28	60.39 (41.43)	30	94.27 (32.85)
IQ	PIQ	105	106.18 (11.73)	16	96.38 (17.82)	26	91.77 (14.59)	27	84.41 (21.22)
		337	108.93 (12.24)	18	97.17 (17.71)	46	92.41 (13.44)	29	88.48 (15.65)

Due to the small number of individuals ≤ 21 years old, the statistical power of our sample was limited when stratifying the cohort by both ASD and LI diagnoses. Although our standard measures used normative samples not exceeding 21 years old, there was no evidence of ceiling effects in this sample when administered to ages beyond the normative data. Therefore, we also assessed group differences among the entire cohort regardless of participant age among the four diagnostic groups: (1) ASD-only (*n* = 24), (2) LI-only (*n* = 53), (3) ASD + LI (*n* = 37), and (4) unaffected (*n* = 369). We also assessed group differences when the cohort was stratified into two ASD diagnostic groups: (1) ASD+ (*n* = 61) and (2) ASD− (*n* = 422). Means and standard deviations for all subtests, IQ, and age for the entire cohort are listed in Table 1.

### Response Dispersion Index

In order to quantify the dispersion of incorrect answers for each diagnostic group, we adapted a measure of item-response distributions from VanMeter and colleagues [11], who utilized an equation for calculating *inefficiency* developed by Hallenbeck et al. (1965) [9]. In this method, *inefficiency* is defined as the product of the sum of the weights of the items missed and the peak item passed.

$$ \mathrm{Inefficiency}=\sum \left(\mathrm{weight}\ \mathrm{of}\ \mathrm{item}\mathrm{s}\ \mathrm{missed}\right)\times \mathrm{peak}\ \mathrm{item}\ \mathrm{passed} $$

However, this measure assumes that test items get progressively more difficult and that testing is discontinued after a traditional ceiling is met. Because many of the language subtests in this study have variable start/stop rules and do not necessarily increase in difficulty in a linear fashion, we used the total number of subtest items instead of the peak item passed. For subtests that did have ceiling rules, any items that were not administered because the subject had already reached a ceiling were counted as incorrect responses. Any items that were not administered because they occurred before the start point for the subject’s age were counted as correct responses. Our modified metric, which we have named the Response Dispersion Index (RDI), therefore is defined as the product of the sum of the weights of the items missed and the total number of subtest items.

$$ \mathrm{RDI}=\sum \left(\mathrm{weight}\ \mathrm{of}\ \mathrm{items}\ \mathrm{missed}\right)\times \#\mathrm{subtest}\ \mathrm{items} $$

We defined the weight of each item missed as the percentage of unaffected family members who correctly answered the test item. For multivariate items (i.e., those in which it is possible to receive partial credit rather than a binary correct/incorrect response), the weight is defined as the sum of the products of the percentage of unaffected family members (≤ 21 years old) who correctly answered the test item and response value for each possible item response. We calculated the RDI for all participants for each CELF and CASL subtest.

$$ \mathrm{Weight}=\sum \limits_{1-n}\left(\%\mathrm{unaffected}\ \mathrm{correct}\ \mathrm{response}\times \mathrm{response}\ \mathrm{value}\right) $$

The weight of each test item decreases as the difficulty level increases, resulting in higher RDI scores for individuals who exhibit a greater degree of within-task variability, as illustrated by our previous hypothetical example (Fig. 1). Individual 1 reached a standard testing ceiling, and the weights for the last 5 items are totaled, resulting in an RDI score of 15. However, despite obtaining the same overall score of 5, individual 2 had a more atypical pattern of incorrect items of varying difficulty (items 2, 4, 7, 9, and 10) and did not reach a testing ceiling. This pattern of incorrect responses results in an RDI score of 23, indicating a greater degree of within-task variability than individual 1 (Fig. 1).

### Rasch person-fit model

**Fig. 2:**
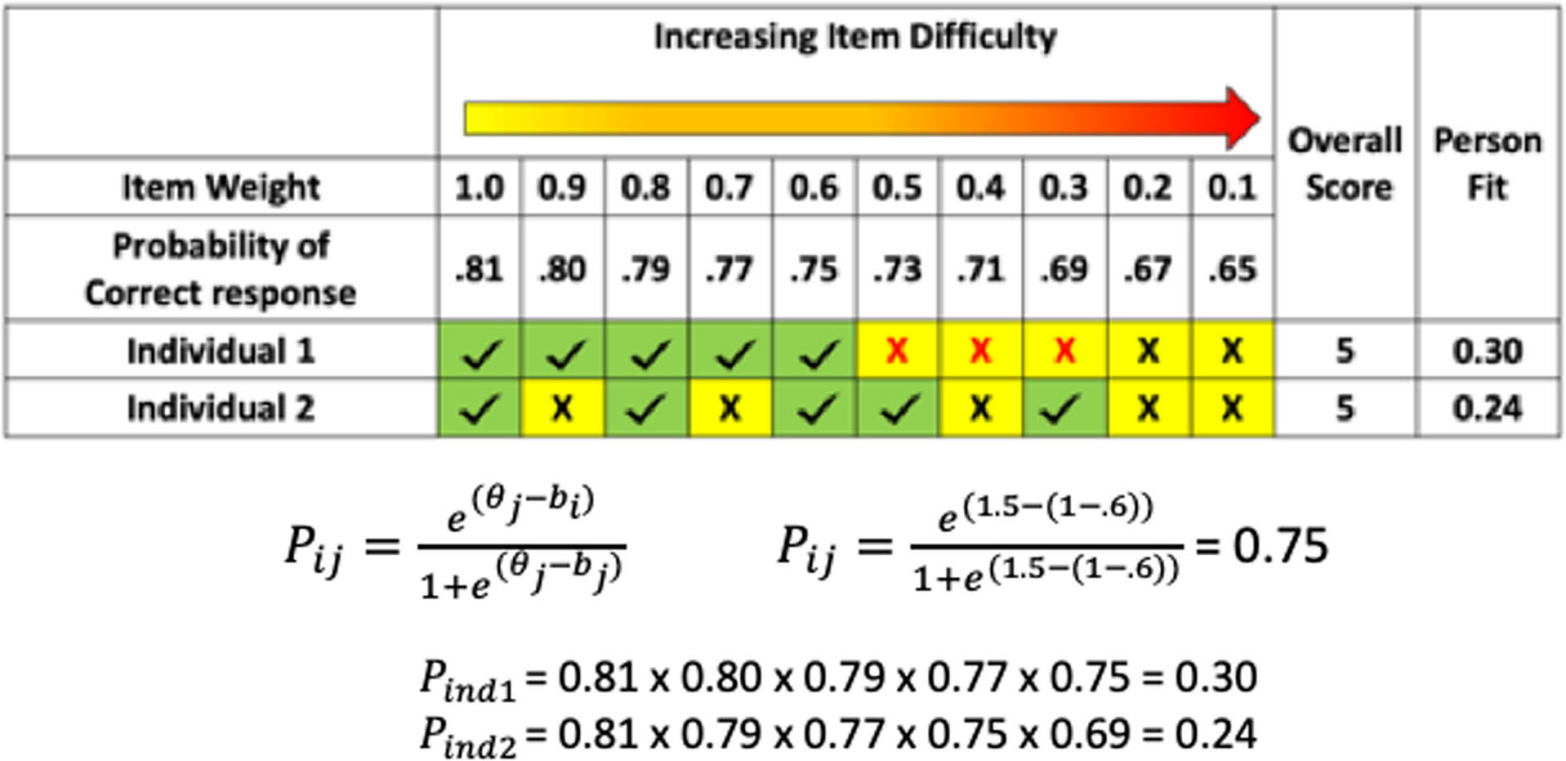
Demonstration of Rasch calculation. Individual 1 represents a participant who displays developmental delay and reaches a testing ceiling (depicted with red X’s). Individual 2 represents a participant who displays developmental difference and does not reach a testing ceiling. The overall score is the total number of correct responses (check marks). Weights for each test item are defined as the percentage of typically developing individuals who responded correctly. A sample calculation for determining the probability of a correct response is shown for item 5, and the overall probability of the observed distribution of responses is shown for individuals 1 and 2

In order to validate our RDI metric, we compared the diagnostic classification accuracy of the RDI with the classification accuracy of a one-parameter Rasch model that is a derivative of the Item Response Theory Model utilized by Godber et al. [8]. This model provides an alternative measure of within-task scatter to assess the degree of dispersion in each language assessment. While other measures of within-task scatter do not take item difficulty into account, the Rasch model calculates the likelihood that the overall subtest score is a true measure of an individual’s ability given the difficulty of the test items the individual answered correctly [8, 38, 39]. It does so by comparing the observed accuracy for each test item to the accuracy expected based on the individual’s overall ability level (Θ) relative to the difficulty of the items that the individual answered correctly. In this case, the difficulty level of each item was defined as the percentage of unaffected individuals who answered the item incorrectly. The model assumes that the overall score of a subtest is indicative of a person’s ability and uses this to predict which weighted test items the individual should have answered correctly. Then, the model assesses the “fit” between this predicted pattern and the individual’s actual pattern of item responses. Since the language tests investigated here assume that individuals with poorer language ability would obtain lower standard scores, a decreased model fit should be indicative of developmental difference. Consider again our hypothetical example in Fig. 2. For each item, the probability of obtaining a correct answer is calculated using the same ability and item difficulty level for both individuals. The person-fit for each individual is then defined as the product of the probability of a correct response for each item that the individual answered correctly. For example, individual 1 answered the first 5 items correctly, so the person-fit metric is the product of the probabilities for the first 5 items, or 0.30. Individual 2, on the other hand, answered items 1, 3, 5, 6, and 8 correctly, so their person-fit score is 0.24, indicating a more atypical response pattern (Fig. 2).

We calculated person-fit for all CELF-4 and CASL subtests using the *psych* package in R [40] and used a Pearson correlation to assess the concordance between RDI and person-fit. While person-fit and RDI were expected to produce similar results, the RDI has several advantages over the Rasch model. The Rasch model assumes that the overall score of a subtest is indicative of a person’s ability and uses this measure to assess the “fit” between this predicted pattern and the individual’s actual pattern of item responses. However, our preliminary analysis indicated that an overall subtest score is not an accurate reflection of a persons’ ability if there is a high degree of within-task scatter. Instead, the RDI eliminates the user-ability parameter (Θ), resulting in a more parsimonious, direct assessment of within-task scatter with fewer assumptions built into the model.

### Statistical methods

In order to standardize RDI scores across subtests, we first calculated aggregate RDI scores across subtests by averaging the standardized residuals (i.e., *z*-scores) of all subtests for each participant. Group differences in subtest standard scores and RDI for all language subtests were assessed using a one-way ANOVA with a post hoc Tukey analysis and Bonferroni correction for multiple testing. Due to the smaller sample size for individuals who were ≤ 21 years old, this analysis was repeated with the entire cohort to increase our statistical power (results shown in Additional file 1). Pearson correlations were used to investigate the relationship between RDI and SRS scores across all diagnostic subgroups. Logistic regression was used to investigate the ability of the RDI to predict ASD diagnosis. Specifically, we tested whether RDI scores on the CASL and CELF-4 can accurately classify participants into ASD+ or ASD− groups. These aggregate RDI values were entered into the model as a predictor variable, with diagnostic classification (ASD+ or ASD−) as the outcome variable and IQ, age, and sex as covariates.

## Results

As a group, the standard scores of individuals with ASD/ASD + LI did not differ significantly from individuals with LI-only for any language measure in the testing battery after correction for multiple testing (Table 1). Despite similar overall standard scores, visual inspection of the distribution of correct/incorrect test item responses appeared to be qualitatively different between the ASD + LI/ASD-only and LI-only groups for a number of measures. This was even more striking when comparing the LI-only group to the ASD + LI group alone.

In order to quantitatively characterize the differences in the item-response distributions between the diagnostic groups, we calculated the RDI for all subtests for each participant. We calculated aggregate RDI scores across subtests by averaging the standardized residuals (i.e., *z*-scores) of all subtests for each participant (Table 2). The ASD+ group had significantly higher RDI scores than ASD− group for all but two subtests (Table 2; Table 3). When the cohort was further subdivided by language impairment, individuals with ASD + LI had higher RDI scores than the LI-only group; however, this difference did not reach statistical significance. This is likely due to the limited power of our small sample size. When considering all individuals regardless of age (Table 4), the ASD + LI group had consistently higher RDI scores than the LI group for all measures of oral language, with the following subtests reaching statistical significance (*p* < .001 unless otherwise noted): CASL Non-literal Language, CASL Pragmatic Judgment, CASL Meaning from Context, CELF Recalling Sentences, CELF Word Definitions, and CELF Word Classes (*p* = .003). While not statistically significant, the ASD + LI group also exhibited consistently higher RDI scores for the remaining measures of language ability (Table 4). This trend was also consistent among individuals without LI. When compared to the unaffected group, the ASD-only group had significantly higher RDI scores (*p* < .001 unless otherwise noted) on the following subtests: CASL Ambiguous Sentences, CASL Pragmatic Judgment, CASL Non-literal Language, CASL Meaning from Context, CELF Concepts and Following Directions, CELF Word Classes (*p* = .002), CELF Formulated Sentences (*p* = .002), and CELF Recalling Sentences (Table 4).

**Table 2:** Group differences in Response Dispersion Index (RDI) for ASD+ and ASD− for participants ≤ 21 years old

		ASD+		ASD−		ASD+ vs ASD−	
		*N*	Mean (SD)	*N*	Mean (SD)	*t*	*p* value
CELF	Concepts and Following Directions	17	.456 (1.170)	81	− .096 (.941)	− 2.103	0.038
	Formulated Sentences	38	.409 (1.206)	124	− .125 (.897)	− 2.527	0.015
	Recalling Sentences	40	.691 (1.244)	128	− .216 (.802)	− 4.340	< 0.001
	Word Structure	13	.549 (1.020)	40	− .178 (.938)	− 2.377	0.021
	Word Definitions	16	.468 (1.307)	46	− .163 (.826)	− 1.809	0.086
	Word Classes–Total	26	.527 (1.185)	86	− .159 (.884)	− 3.193	0.002
CASL	Ambiguous Sentences	14	.582 (1.067)	60	− .136 (.942)	− 2.505	0.015
	Inference	17	.889 (1.189)	74	− .204 (.835)	− 3.594	0.002
	Pragmatic Judgment	35	.550 (1.058)	122	− .158 (.928)	− 3.850	< 0.001
	Non-literal Language	27	.579 (1.163)	94	− .166 (.887)	− 3.085	0.004
	Meaning from Context	12	.446 (1.092)	60	− .089 (.966)	− 1.715	0.091
	Average RDI all subtests	44	.775 (1.058)	133	− .063 (.763)	− 4.850	< 0.001

**Table 3:** Group differences in Response Dispersion Index (RDI) for ASD+ and ASD− for all participants

Test	Subtest	ASD+		ASD−		ASD+ vs ASD−	
		*N*	Mean (SD)	*N*	Mean (SD)	*t*	*p* value
CELF	Concepts and Following Directions	30	.419 (1.079)	93	− .131 (.943)	− 2.649	.009
	Formulated Sentences	48	1.057 (1.429)	377	− .135 (.844)	− 5.652	< .001
	Recalling Sentences	52	1.363 (1.454)	394	− .180 (.761)	− 7.519	< .001
	Word Structure	17	.450 (.987)	50	− .153 (.967)	− 2.210	.031
	Word Definitions	22	1.613 (1.830)	300	− .118 (.796)	− 4.406	< .001
	Word Classes-Total	34	1.486 (1.395)	340	− .149 (.817)	− 6.716	< .001
CASL	Ambiguous Sentences	23	1.296 (1.076)	307	− .097 (.925)	− 6.883	< .001
	Inference	26	.956 (1.032)	80	− .311 (.770)	− 5.759	< .001
	Pragmatic Judgment	53	1.240 (1.168)	388	− .169 (.845)	− 8.486	< .001
	Non-literal Language	36	1.601 (1.368)	341	− .169 (.783)	− 7.630	< .001
	Meaning from Context	20	1.862 (1.478)	305	− .122 (.828)	− 5.943	< .001

**Table 4:** Group differences in Response Dispersion Index (RDI) for phenotypic groups for all participants

Test		Unaffected		ASD-only		LI-only		ASD + LI			Omnibus ANOVA *p* value	Unaffected vs ASD-only *p* value	LI-only vs LI + ASD *p* value
		*N*	Mean (SD)	*N*	Mean (SD)	*N*	Mean (SD)	*N*	Mean (SD)	*F*			
CELF	Concepts and Following Directions	68	− .413 (.723)	9	− .096 (1.089)	25	.638 (1.050)	21	.651 (1.016)	13.180	< .001	< .001	1.000
	Formulated Sentences	331	− .293 (.604)	18	.418 (1.295)	46	1.006 (1.327)	30	1.440 (1.386)	69.426	< .001	0.002	0.112
	Recalling Sentences	347	− .331 (.568)	20	.483 (1.213)	47	.935 (1.040)	32	1.913 (1.328)	121.291	< .001	< .001	< .001
	Word Structure	34	− .548 (.610)	6	− .044 (.926)	16	.688 (1.062)	11	.719 (.951)	11.608	< .001	0.512	1.000
	Word Definitions	277	− .247 (.596)	9	.081 (.656)	23	1.434 (1.188)	13	2.674 (1.611)	101.749	< .001	0.533	< .001
	Word Classes-Total	310	− .291 (.618)	13	.391 (.719)	30	1.327 (1.120)	21	2.163 (1.281)	114.278	< .001	0.005	< .001
CASL	Ambiguous Sentences	283	− .224 (.825)	10	.744 (1.019)	24	1.404 (.696)	13	1.721 (.947)	51.376	< .001	0.002	0.684
	Inference	68	− .515 (.559)	7	.269 (1.047)	12	.849 (.794)	19	1.210 (.929)	37.411	< .001	0.029	0.504
	Pragmatic Judgment	341	− .298 (.691)	22	.829 (1.248)	47	.760 (1.212)	31	1.532 (1.031)	71.767	< .001	< .001	< .001
	Non-literal Language	306	− .300 (.593)	14	.558 (.966)	35	.977 (1.214)	22	2.265 (1.165)	111.968	< .001	< .001	< .001
	Meaning from Context	280	− .236 (.708)	9	.898 (1.220)	25	1.151 (1.013)	11	2.652 (1.199)	74.799	< .001	< .001	< .001

In order to validate our findings, we used a Rasch person-fit model as an assessment of within-task variability. As described earlier, this model calculates the likelihood that the overall subtest score is a true measure of an individual’s ability given the difficulty of the test items the individual answered correctly [8, 38, 39]. Since the language tests investigated here assume that individuals with poorer language ability would obtain lower standard scores, a decreased model fit should be indicative of greater within-task variability. While this procedure is comparable to our RDI metric, by incorporating the individual ability level, the person-fit model includes a measure of developmental delay to infer within-test scatter. The person-fit metric was highly correlated with RDI (*r* range − 0.84 to − 0.98; *p* < .001) for all subtests of the CASL and all but one subtest of the CELF-4 (≤ 21 year old group shown in Table 5; entire cohort listed in Table 6; scatterplots shown in Additional file 1: Figure S1), providing a source of convergent validity for the RDI metric results.

**Table 5:** Rasch model of developmental difference for CELF-4 and CASL subtests and correlation with Response Dispersion Index (RDI) for participants ≤ 21 years old

		ASD+ person-fit	ASD− person-fit	ASD+ vs. ASD−	Pearson’s correlation person-fit vs RDI		
Test	Subtest	Mean (SD)	Mean (SD)	*p* value	*N*	*r*	*p* value
CELF	Concepts and Following Directions	.53 (.231)	.73 (.255)	0.004	98	− 0.263	0.008
	Formulated Sentences	.48 (.294)	.67 (.281)	< 0.001	162	− 0.938	< 0.001
	Recalling Sentences	.47 (.290)	.68 (.240)	< 0.001	168	− 0.983	< 0.001
	Word Structure	.48 (.256)	.66 (.235)	0.022	52	− 0.994	< 0.001
	Word Definitions	.36 (.207)	.55 (.216)	0.005	61	− 0.814	< 0.001
	Word Classes-Total	.54 (1.235)	1.37 (1.173)	0.004	110	− 0.983	< 0.001
CASL	Ambiguous Sentences	.29 (.266)	.45 (.259)	0.038	74	− 0.980	< 0.001
	Inference	.34 (.248)	.57 (.206)	< 0.001	90	− 0.981	< 0.001
	Pragmatic Judgment	.02 (.021)	.04 (.028)	< 0.001	160	− 0.861	< 0.001
	Non-literal Language	.29 (.266)	.45 (.259)	0.004	120	− 0.981	< 0.001
	Meaning from Context	.35 (.226)	.47 (.231)	0.105	71	− 0.975	< 0.001

**Table 6:** Rasch model of developmental difference for CELF-4 and CASL subtests and correlation with Response Dispersion Index (RDI) for all participants

Test	Subtest	ASD+ person-fit	ASD− person-fit	ASD+ vs. ASD−	Pearson’s correlation person-fit vs RDI		
		Mean (SD)	Mean (SD)	*p* value	*N*	*r*	*p* value
CELF	Concepts and Following Directions	.57 (.279)	.71 (.252)	0.010	123	− 0.998	< .001
	Formulated Sentences	.45 (.297)	.80 (.232)	< .001	425	− 0.945	< .001
	Recalling Sentences	.44 (.281)	.79 (.209)	< .001	446	− 0.947	< .001
	Word Structure	.49 (.238)	.64 (.233)	0.026	67	− 0.994	< .001
	Word Definitions	.30 (.216)	.59 (.207)	< .001	322	− 0.773	< .001
	Word Classes-Total	.45 (.267)	.78 (.185)	< .001	374	− 0.996	< .001
CASL	Ambiguous Sentences	.21 (.242)	.57 (.271)	< .001	330	− 0.984	< .001
	Inference	.26 (.261)	.57 (.216)	< .001	106	− 0.983	< .001
	Pragmatic Judgment	.43 (.290)	.82 (.233)	< .001	441	− 0.997	< .001
	Non-literal Language	.32 (.275)	.72 (.202)	< .001	377	− 0.992	< .001
	Meaning from Context	.27 (.254)	.67 (.211)	< .001	325	− 0.989	< .001

Given the group differences between the ASD+ and ASD− groups, we next investigated the relationship between RDI and quantitative autistic traits across all individuals, regardless of diagnosis, as measured by the SRS. We found that SRS scores were positively correlated with RDI scores on all subtests except for the CELF-4 Word Structure subtest (Table 7). This was also true when considering all individuals in the NJLAGS cohort (Table 7; scatterplots shown in Additional file 1: Figure S2). In addition to correlation with SRS scores, we examined the accuracy of the RDI in predicting ASD diagnosis using a logistic regression analysis. The aggregate RDI values were entered into the model as a predictor variable, with ASD diagnostic classification as the outcome variable. IQ, age, and sex were included as covariates. This model showed that the RDI was able to successfully predict ASD diagnosis among individuals ≤ 21 years old 81% of the time (Hosmer-Lemeshow goodness of fit: *χ*^2^ (1) = 2.518, df = 8, *p* = .961; Nagelkerke *R*^2^ = .379). Aggregate RDI contributed significantly to the classification accuracy of the model (*B*(S.E.) = 1.359 (0.334), *Z*_wald_ = 16.569, *p* < .001), with the odds ratio (Exp(*B*) = 3.893, 95% CI [2.023, 7.491]) indicating that as RDI increases by one standard deviation, participants were approximately 3.8 times as likely to be diagnosed with ASD.

**Table 7:** Positive correlation between Response Dispersion Index (RDI) and Social Responsiveness Scale (SRS)

		< 21 years old			All participants		
		*N*	Correlation	*p* value	*N*	Correlation	*p* value
CELF	Concepts and Following Directions	69	0.381	0.001	123	0.358	0.001
	Formulated Sentences	117	0.251	0.006	428	0.344	< 0.001
	Recalling Sentences	121	0.317	< 0.001	450	0.388	< 0.001
	Word Structure	33	0.256	0.151	67	0.312	0.044
	Word Definitions	47	0.398	0.006	323	0.431	< 0.001
	Word Classes-Total	86	0.325	0.002	377	0.381	< 0.001
CASL	Ambiguous Sentences	57	0.438	0.001	334	0.322	< 0.001
	Inference	72	0.374	0.001	108	0.484	< 0.001
	Pragmatic Judgment	115	0.303	0.001	443	0.373	< 0.001
	Non-literal Language	94	0.320	0.002	386	0.409	< 0.001
	Meaning from Context	60	0.280	0.030	329	0.407	< 0.001

## Discussion

Although delays in various aspects of development are routinely quantified and treated as continuously distributed variables, non-sequential skill acquisition resulting in *developmental deviance* or *developmental difference* has conventionally been treated as a qualitative, dichotomous descriptor of atypical skill profiles. In this study, instead of using a categorical approach, we developed a measure of the dispersion of item-level responses called the RDI, which is modified from VanMeter and colleagues’ *inefficiency* metric, to determine the degree of within-task variability in language skills [11]. This pilot study is the first, to our knowledge, to use a quantitative metric to investigate within-task variability of performance on standardized language tests in individuals with ASD [11].

Using the RDI, we demonstrated that individuals with ASD exhibit a higher degree of within-task variability than individuals without ASD, and individuals with ASD + LI exhibited the highest levels of within-task variability for all language measures. The RDI was positively correlated with quantitative autistic traits (as measured by the SRS) across all members of the NJLAGS cohort regardless of ASD/LI diagnosis. We also demonstrated that the RDI is a strong predictor of ASD status, supporting previous findings that atypical, non-sequential development is associated with ASD [11, 13, 27, 41]. These findings suggest that quantifying within-task variability in language development based on item-level subtest response patterns adds important information about language in ASD that is not captured by standard performance summary scores. We have demonstrated that the RDI can be used effectively to quantify atypical, non-sequential development.

While the RDI generally differed between individuals with LI only and those with ASD across language measures, the Word Structure subtest from the CELF-4 was one exception. The RDI calculated for this subtest did not reach significance between diagnostic groups and was also the only subtest that was not significantly correlated with quantitative ASD traits across all groups. Although this could be due to the smaller sample size for this subtest, Word Structure involves a cloze procedure in which the child must complete a sentence with a targeted structure (morphology) [33]. Morphological structures assessed with this subtest include verb tense, copula and auxiliary forms, referential pronouns, and other grammatical morphemes. While there is a fairly robust literature documenting differences in the development of semantics, pragmatics, and syntax in individuals with ASD [12–15], evidence for atypical development in word-level morphology in ASD is sparse. One study found that morphological skills in children with ASD, while impaired relative to typical children, were similar to children with developmental delay [42]. Furthermore, the researchers found few differences in the order of acquisition of specific grammatical morphemes between the children with ASD and typical children. Thus, the lack of association between RDI in morphology and ASD diagnosis in the present study is consistent with previous findings that the development of morphology in children with ASD, while delayed, is not necessarily acquired in an atypical sequence.

Although this is the first study to use a quantitative metric to assess within-task variability in direct language testing, clinicians have been interested in inconsistent or unusual item-level response patterns within a test or subtest as a diagnostic variable or marker of cognitive dysfunction for many years [7–10, 28]. Similar to clinical language measures, psychometric measures of cognition assume a fixed order in skill development and, as such, are often arranged hierarchically. This assumption makes these tools effective in identifying developmental delay; however, as demonstrated by Visser et al., this methodology may under-characterize the extent of within-task variability [7]. The RDI metric used in our study eliminates this assumption by calculating the difficulty of individual test items. The weight of each test item decreases as the difficulty level increases, regardless of test item order, making the RDI metric a robust method for detecting developmental difference for a variety of assessment types.

An alternative metric for detecting within-task variability is the Rasch person-fit model based on Item Response Theory. Godber et al. successfully used this method to discriminate between children treated with cranial irradiation for acute lymphoblastic leukemia and matched healthy controls [8]. As described earlier, Rasch person-fit is a metric that estimates an individual’s overall ability level by analyzing the difficulty level of correctly answered items within a psychometric assessment. The difficulty level of each item is defined as the percentage of unaffected individuals (or a normative sample, if available) who answer the item correctly. This procedure is comparable to our RDI metric, and we found that the person-fit metric was highly correlated with the RDI when applied to the language measures in the NJLAGS sample.

While person-fit and RDI produce similar results, the RDI has several advantages over the Rasch model of intra-subtest scatter. The Rasch model assumes that the overall score of a subtest is indicative of a person’s ability and uses this measure to predict which weighted test items that individual should have answered correctly, assessing the “fit” between this predicted pattern and the individual’s actual pattern of item responses. In contrast, the RDI metric makes no mathematical assumptions regarding the relationship between an overall subtest score and a person’s ability. Furthermore, by eliminating the user-ability parameter, the RDI directly measures within-task scatter, making this metric more parsimonious and reducing the number of assumptions built into the model. This is particularly important given that the user-ability parameter is typically defined by patterns of developmental delay, not difference. In the present study, we demonstrate that developmental delay fails to adequately capture all patterns of atypical response profiles. Additionally, by using a normative sample to define item weights, the RDI can be standardized for a given psychometric assessment, making it a tool that could be adapted for use in clinical settings to provide additional information about an individual’s language ability without requiring any further testing. Current and newly developed language tests could easily provide clinicians with standardized RDI scores, in addition to indices of delay, as part of an automated scoring software program.

It is important to note that the RDI does not represent a correlation between individual item responses and the degree of within-task scatter; rather, the RDI quantifies the distribution of all incorrect responses as a group. Since developmental difference is characterized by an atypical sequence of developmental milestone attainment, it is more informative to assess the overall item-response pattern as opposed to individual item responses within a given task.

### Limitations

Since the NJLAGS project was not originally designed for this type of pilot study, there are a few limitations to using this dataset for our study. First, the NJLAGS sample was ascertained for multiplex families to increase the genetic loading for language impairment in these families. As such, NJLAGS may represent a more severe language impairment cohort and may not be representative of other ASD or language cohorts. Second, as this is a retrospective study of the NJLAGS cohort, our diagnostic groups were not matched for age or IQ. However, our statistical analyses indicate that age and IQ do not have a significant effect on the relationship between ASD traits and RDI. Lastly, the weights utilized by the RDI metric were derived using the unaffected group, not an independent normative sample. Future studies are needed to standardize RDI using an unselected normative sample to define item weights. Ideally, RDI weights would be calculated at the time of development of new or revised language measures using the original standardization sample.

Another potential limitation is that the language tests used with this cohort were administered using standard basal and ceiling rules. While this is standard clinical procedure, we might expect greater variability in RDI if each subtest was administered in its entirety, since individuals exhibiting developmental difference (vs. delay) might be more likely to answer earlier items incorrectly and more difficult items beyond the ceiling correctly. However, the downside to administering the entire subtest is the amount of time it takes, limiting clinical feasibility. Since we found significant differences regardless, it is likely that the RDI is robust enough to provide clinically useful information even while following standard basal and ceiling rules. However, it may be worthwhile for future studies to investigate the impact of administering entire subtests on RDI rather than following basal and ceiling rules.

## Future directions

Standardized tests have been criticized because performance summary scores do not take the individual’s pattern of the responses into account. The results of this pilot study suggest that further research is warranted to determine whether developmental difference, as measured by the RDI, is a meaningful indicator of dysfunction that is not captured by standard performance summary scores. If the association between language RDI and ASD is confirmed, it may have implications for screening, diagnosis, and advancing the understanding of language development in ASD. It would be valuable to follow children with LI, both with and without ASD, longitudinally to determine if RDI scores correlate with long-term outcomes and thereby inform prognostication. While this has never been studied specifically, there is some evidence that developmental difference may predict outcomes for children. For example, in the area of speech sound disorders, surface error patterns may reflect different underlying phonological processing deficits, which in turn may lead to divergent language and literacy outcomes. Several longitudinal studies demonstrate that children who initially present with non-developmental speech errors have poorer phonological awareness, decoding, spelling, and reading comprehension scores at follow-up compared to children who presented initially with developmentally delayed, but typical, speech sound error patterns [43, 44]. It is likely that certain types of tests will be more informative than others, and the importance of factors such as age and ability level will require exploration.

Although this study addresses only difference in performance on certain language measures and its role in ASD, the concept may apply to cognitive tests and other measures of development and should be evaluated in other neurodevelopmental disorders and across diagnostic categories. Intra-subtest scatter on IQ tests, for example, has been found to be associated with cognitive inefficiency and variability in attention or arousal, among other things, and there is evidence that it may be useful for detecting cognitive dysfunction among individuals with relatively normal profiles on IQ tests [8, 28]. The RDI may also be useful for identifying subtle but meaningful dysfunction associated with attention-deficit hyperactivity disorder (ADHD), specific learning disorders, and acquired insults such as traumatic brain injury and medical interventions such as chemotherapy, radiation therapy, and early anesthesia exposure.

While it is beyond the scope of the present study, future research is warranted to explore the underlying reasons for the atypical profile of language skills seen in individuals with ASD that is quantified by the RDI. Uneven response patterns may correlate with specific features that are commonly seen in individuals with ASD, such as differences in executive functioning [45], motivation [46], theory of mind [47], and repetitive behaviors [48]. Such features likely impact language acquisition itself as well as the ability to perform under standardized conditions, the latter of which would be influenced by task demands specific to individual language measures (e.g., working memory requirements, complexity of verbal directions). Examining the correlation between quantitative traits in general cognitive processes and RDI in language measures may shed light on which mechanisms hinder or promote efficiency in language acquisition. Similarly, comparing task demands and response patterns may identify certain features of tests themselves that are likely to influence an individual’s performance.

## Conclusions

We investigated within-task variability in language skills using a quantitative, continuous metric rather than treating difference as a qualitative, dichotomous trait. This metric, the RDI, was correlated with ASD traits among the entire NJLAGS cohort studied, supporting a continuous model of developmental difference. The RDI can also be adapted to other clinical measures to investigate the degree of difference in various developmental domains; such analyses could help obtain a more comprehensive developmental profile of individuals with ASD and other neurodevelopmental disorders, which in turn might be used to predict long-term outcomes.

## Additional file

Additional file 1: **Figure S1.** Correlation between RDI and person-fit for all subtests. **Figure S2.** Correlation between RDI and SRS for all subtests. (DOCX 25986 kb)

## Data Availability

The datasets generated and/or analyzed during the current study are available in the NIMH Data Archive repository, https://ndar.nih.gov/edit_collection.html?id=1932.

## Abbreviations

ASD: Autism spectrum disorder
ASD−: No diagnosis of autism spectrum disorder
ASD+: Diagnosis of autism spectrum disorder
ASD + LI: Autism spectrum disorder and language impairment
ASD-only: Affected for autism spectrum disorder but not language impairment
CASL: Comprehensive Assessment of Spoken Language
CELF-4: Clinical Evaluation of Language Fundamentals, Fourth Edition
DLD: Developmental language disorder
IQ: Intelligence quotient
LI: Language impairment
LI-only: Affected for language impairment but not autism spectrum disorder
NJLAGS: New Jersey Language and Autism Genetics Study
RDI: Response Dispersion Index
SLI: Specific language impairment
SRS: Social Responsiveness Scale
