# Supplementary material for: Within-task variability on standardized language tests predicts autism spectrum disorder: a pilot study of the Response Dispersion Index
Source: J Neurodev Disord. 2019 Sep 13;11:21. doi: 10.1186/s11689-019-9283-z (PMC6744656; doi:10.1186/s11689-019-9283-z)

Additional file 1: Figure S1: Correlation between RDI and person-fit for all subtests


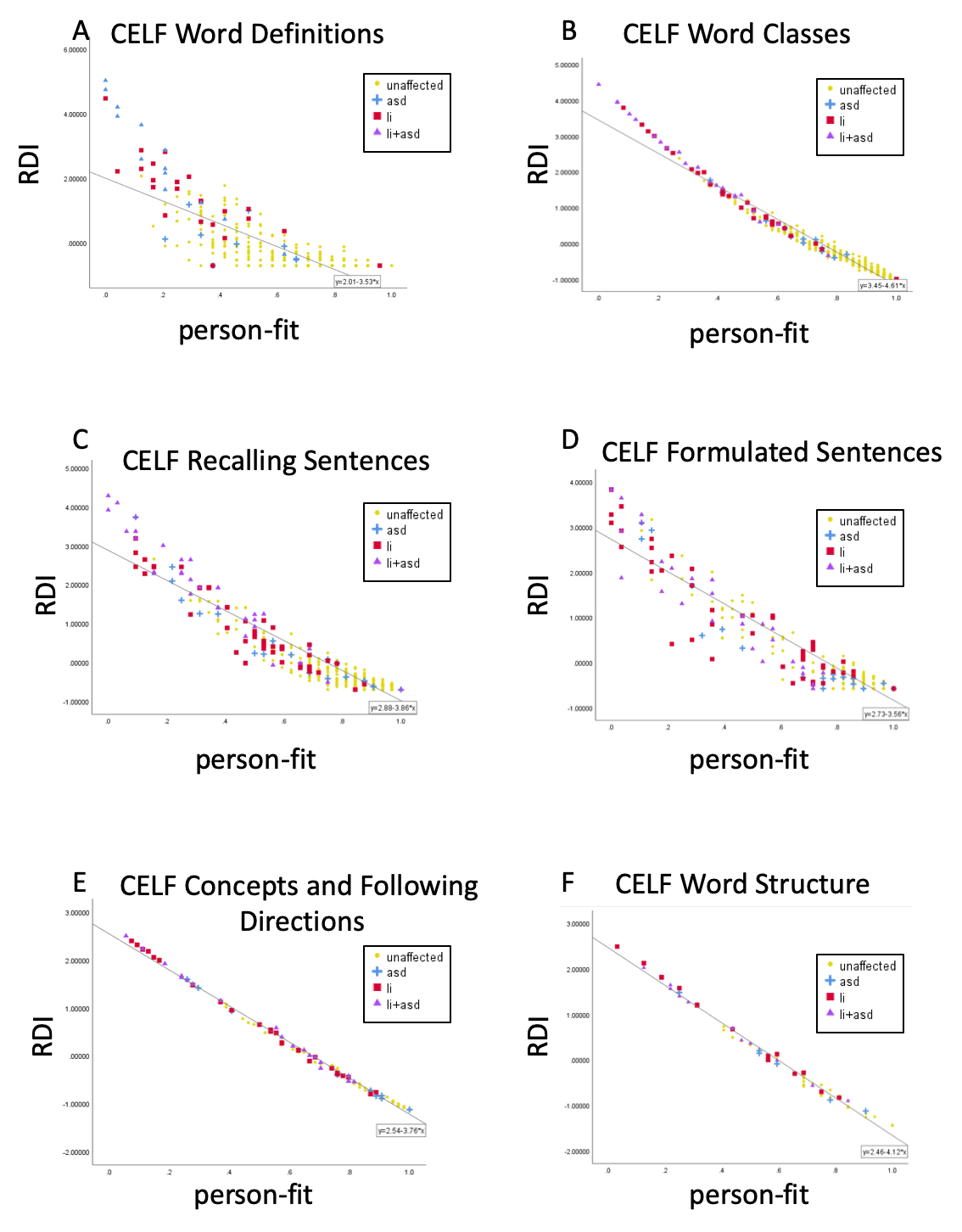


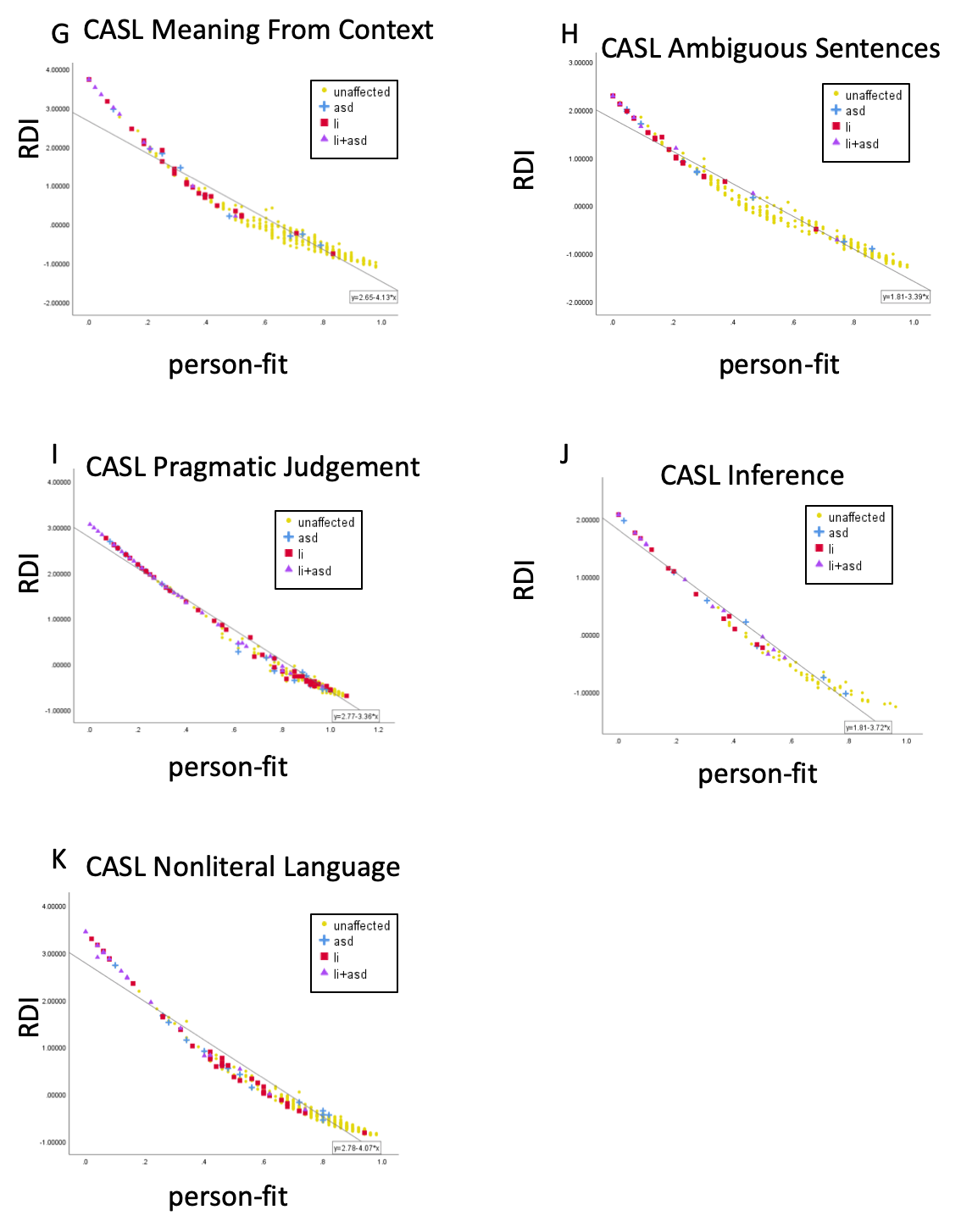


Additional file 1: Figure S2: Correlation between RDI and SRS for all subtests


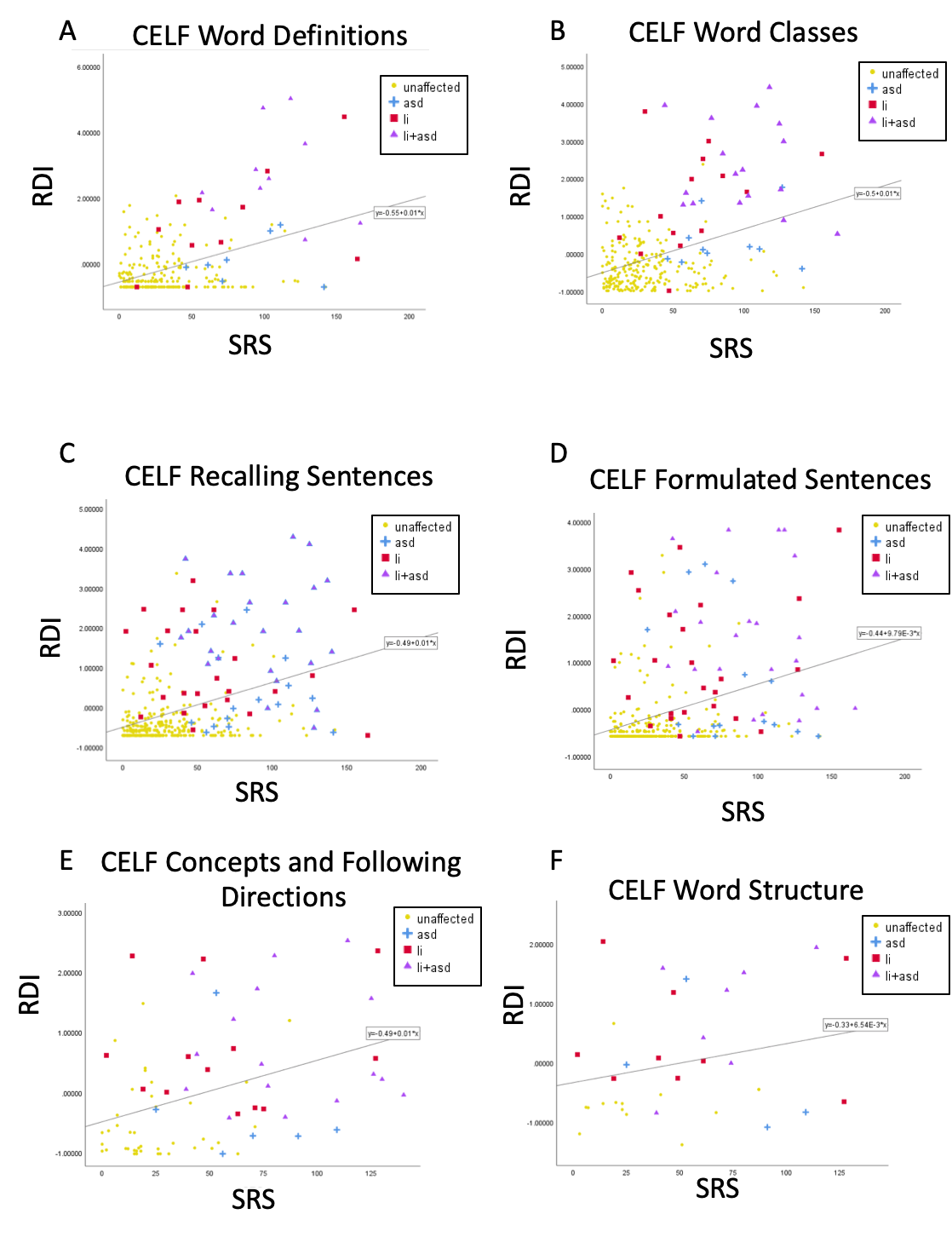


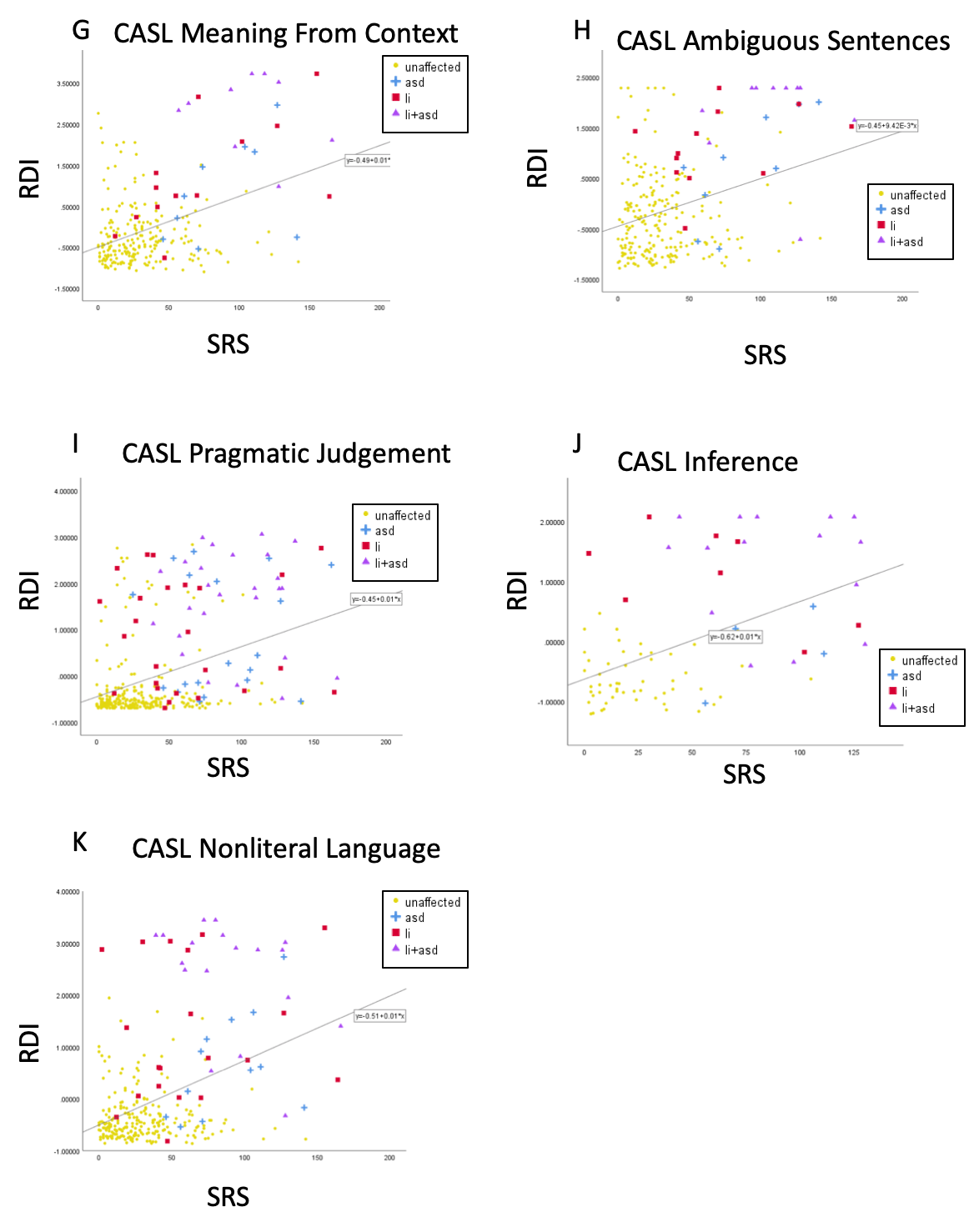

Supplement: Supplementary file 1 — Figure S1. Correlation between RDI and person-fit for all subtests. Figure S2. Correlation between RDI and SRS for all subtests. (DOCX 25986 kb) [file 11689_2019_9283_MOESM1_ESM.docx]
